# The Wards as a Patient Sees Them

**Published:** 1920-04-03

**Authors:** 


### A HOSPITAL FROM INSIDE.

### The Wards as a Patient Sees Them.

Under this title comes to hand a small eight-page pamphlet which portrays in an unusually realistic manner the wards of a hospital as the patient sees them. The author gives us the results of his own reflections during the month's stay as a patient in St. Bartholomew's, and though, as he says, there is a great factor of happiness for staff and patient alike in their recognition of the others' way of looking at things, and that in his own writing he can only set down with certainty a single patient's outlook; yet we must confess he has drawn a picture of a view which, while intensely appreciative of all that our hospitals do, is one which, we trust, will be read by the many thousands of one-time hospital patients and bring home to them their obligation to the voluntary hospital system.

### The Ordinary Flow of Existence.

Many have left their homes, perhaps, in the morning without the least suspicion that anything would interrupt the ordinary regular flow of existence, supposing that they would conduct their business in the usual way. Yet a few hours later they have found themselves in one of the fifteen or so beds of a hospital ward, unable to move, carefully tended but shorn even of their own name, and become No. ——— of A ——— ward.

Many, indeed, have had this experience, and in future yet more will occupy these beds, and if they do not learn fully to appreciate the wonders of voluntary hospital organisation, the magnificent, untiring energy of the staff as a whole, and particularly of the nurses who care for them, then we ask them to read this small pamphlet, which, though referring primarily to the great city hospital, mirrors with extraordinary clearness the atmosphere of them all.

### A Home Comfort.

If, too, it happens that the reader finds himself among that 90 per cent. of the population who have never given direct or indirect support, great or small, towards support of a voluntary hospital, he may feel, with the author, that acute twinge, as he realises, as in the case of Bart's for example, that the very nurses whom he would do so much to repay, must still go for their rest and home to a curious huddle of ancient houses, with narrow passages and uninteresting rooms, and on the edge of a busy City street, down which actually he has often passed. And yet only funds are needed to improve all this and to give them a home and comfort. Funds which would readily materialise if all Numbers ——— of A ——— ward were themselves to give a little.

The author-patient says, "It ought to be done, it has got to be done, and us is the chaps to do it." Many are the people who might thus summarise their bounden duty to the poverty of the voluntary hospitals.

